# Expanding the Spectrum of Rearrangements Involving Chromosome 19: A Mild Phenotype Associated with a 19p13.12–p13.13 Deletion

**DOI:** 10.1002/ajmg.a.35254

**Published:** 2012-03-14

**Authors:** Giuseppe Marangi, Daniela Orteschi, Federico Vigevano, Jillian Felie, Christopher A Walsh, M Chiara Manzini, Giovanni Neri

**Affiliations:** 1 Institute of Medical Genetics, Università Cattolica del Sacro CuoreRoma, Italy; 2 Division of Neurology, “Bambino Gesù” Children's Hospital IRCCSRoma, Italy; 3 Division of Genetics, Manton Center for Orphan Diseases and Howard Hughes Medical Center, Children's Hospital BostonBoston, Massachusetts

**Keywords:** 19p13.12–p13.13 deletion, intellectual disability, attention deficit hyperactivity disorder, phenotypic variability

## Abstract

We report on a patient with a 1.2 Mb 19p13.12–p13.13 deletion. Compared to previously reported individuals with partially overlapping deletions, the propositus presented with a less severe phenotype, consisting of mild intellectual disability and behavior anomalies, with episodes of simple febrile seizures and without significant physical anomalies or major malformations. The deleted region includes 29 coding genes, some of which have already been demonstrated to be involved in cognitive processes. Mutations in two of them, *CC2D1A* and *TECR*, were recently reported to be responsible for non-syndromal, autosomal recessive intellectual disability. The residual alleles of all of these genes were submitted to sequence analysis. No sequence variants were found that could be considered pathogenic. This patient constitutes a further example of the wide phenotypic variability associated with chromosomal rearrangements, likely due to the different size of deleted/duplicated segments. © 2012 Wiley Periodicals, Inc.

## INTRODUCTION

Rearrangements involving chromosome 19 constitute a rare finding. However, for a few restricted regions, a number of patients have been reported, sufficient to delineate recognizable phenotypes [Malan et al., 2009]. Recently, Bonaglia et al. [2010] reviewed and compared four patients, carriers of 19p13.12–p13.13 microdeletions, with a region of overlap of about 360 kb, and identified recurrent clinical features, like intellectual disability (ID), speech and motor delay, hearing impairment, brachycephaly, anteverted nares, and external ear anomalies.

Here, we describe a boy with a 1.2 Mb 19p13.12–p13.13 deletion including the 360 kb region of Bonaglia et al. [2010], who presented with different and milder phenotypic traits. The possible pathogenic role of candidate genes encompassed by the microdeletion was evaluated by sequence analysis.

## CLINICAL REPORT

The propositus was the only child of healthy, nonconsanguineous Caucasian parents, who were 34 (father) and 30 years old (mother), respectively, at the time of conception. He was delivered by cesarean at 38 weeks of a pregnancy complicated by premature aging of placenta during the last 3 weeks of gestation. Birth-weight was 2470 g (between 3rd and 10th centile), length 48 cm (25th centile), and head circumference 33.5 cm (50th centile). Apgar scores were 9 and 10, at 1 and 5 min, respectively. No medical concerns were raised at birth.

Psychomotor milestones were slightly delayed: The child was able to walk without support at 17 months and spoke his first words at 30 months. At 8 years 5 months the evaluation with the non-verbal intelligence scale Leiter-R showed an IQ of 58, with significant attention deficit.

The child had three episodes of febrile seizures, the first two during the 4th year of life, the third when he was 7 years old. Several EEG recordings showed epileptiform focal abnormalities, mainly occipital, activated by sleep. A susceptibility to respiratory tract infections was reported. Family history revealed that also a female, first cousin on the father's side, was affected with ID. Two brain MRIs were normal. Ophthalmological and audiometric evaluation (both performed at age 6) gave normal results. Cardiac clinical assessment did not show any heart anomaly.

At age 3 years 10 months his measures were: Weight 15.5 kg (25th–50th centile), height 98 cm (25th centile), and head circumference 49 cm (10 th centile). He had not yet reached complete control of urinary sphincters and suffered from mild constipation.

At our last evaluation he was 7 years 3 months old, weighed 24 kg (50th centile), had a stature of 119 cm (25th centile) and a head circumference of 50.7 cm (10th–25th centile).

He presented with facial features that showed strong family resemblance, with few minor anomalies, such as upsweep of the frontal hairline, small ears with prominent anti-helix, mild hypertelorism, and highly arched palate. Mild generalized hypertrichosis was noted. He had clinodactyly of 5th finger bilaterally, regular palmar creases, a dermatoglyphic pattern with four whorls and one arch (right hand: W, A, L^u^, W, W; left hand: L^u^, W, L^u^, W, L^u^) and crowding of toes. External genitalia were normal male with bilaterally descended testes. Heart sounds were normal on auscultation.

At that time, the boy showed mild ID, the speech being apparently less affected. His behavior was characterized by hyperactivity and attention deficit (ADHD).

## GENETIC TESTS

We performed Array-CGH analysis using Agilent oligonucleotide-array kit 44B (Human Genome CGH Microarray Kit 44B; Agilent Technologies, Santa Clara, CA), with an average resolution of about 75 kb, following the manufacturer's instructions. An interstitial deletion spanning about 1.2 Mb of chromosome region 19p13.12–p13.13 was detected, with distal breakpoint at about 13.5 Mb from the 19p telomere (most centromeric deleted probe A_14_P109213, mapping at 13,471,947 bp from the 19p telomere, most telomeric non-deleted probe A_14_P104509, at 13,424,832 bp) and proximal breakpoint at about 14.7 Mb from the 19p telomere (most centromeric deleted probe A_14_P136346, at 14,683,937 bp, most telomeric non-deleted probe A_14_P108139, at 14,703,837 bp)(Genome Browser Assembly hg18, NCBI 36, March 2006).

Since array-CGH analysis was normal on both parents, we concluded that the deletion was de novo.

**FIG. 1:**
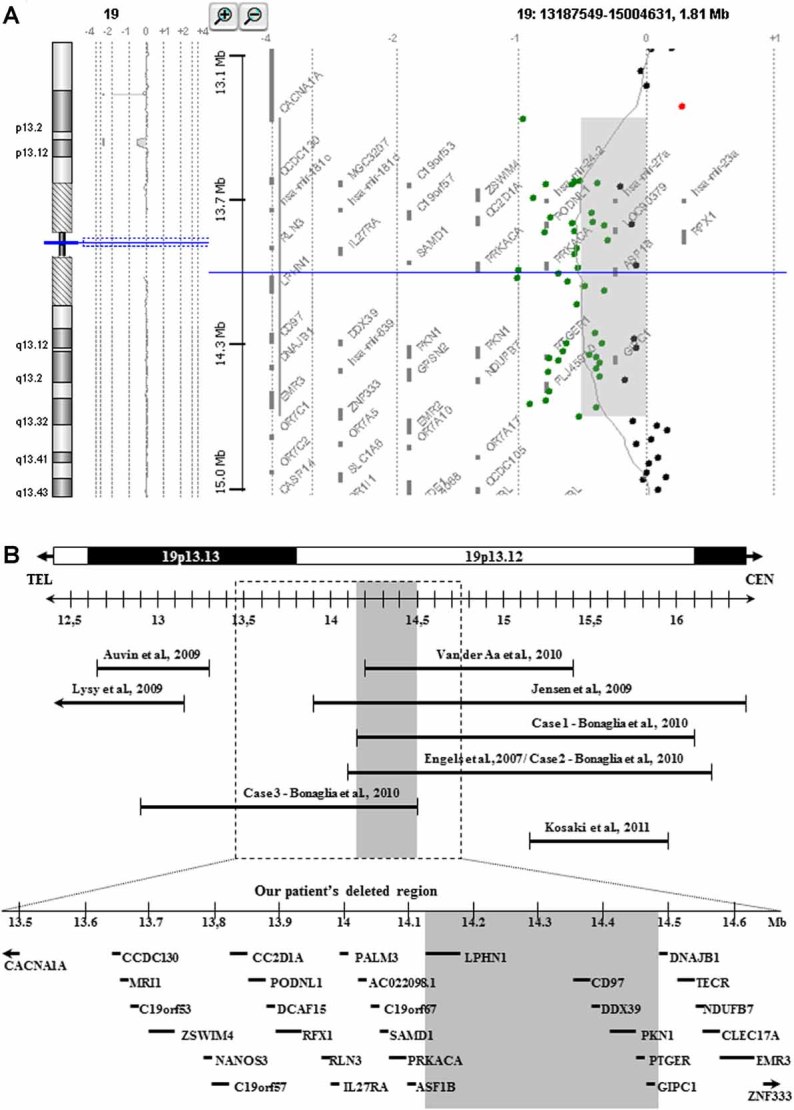
A: Graphical overview of Array-CGH analysis results (Human Genome CGH Microarray Kit 44B; Agilent Technologies, Santa Clara, CA) of our patient, with an average resolution of about 75 kb, showing an interstitial deletion spanning about 1.2 Mb of chromosome region 19p13.12–p13.13, ranging from about 13.5 Mb to 14.7 Mb from the 19p telomere (Genome Browser Assembly hg18, NCBI 36, March 2006). **B**: Schematic representation of 19p13.12–p13.13 region and of reported deletions (horizontal lines). Dashed rectangles mark the boundaries of the region deleted in our patient that is enlarged on the bottom part of the figure, along with the map of included genes. Grey shaded rectangles indicate the 360 kb SRO (the shortest region of overlap) described by Bonaglia et al. [2010].

Included in the deletion interval are the full open-reading frames of 29 protein-coding genes (*CCDC130*, *MRI1*, *C19orf53*, *ZSWIM4*, *NANOS3*, *C19orf57*, *CC2D1A*, *PODNL1*, *DCAF15*, *RFX1*, *RLN3*, *IL27RA*, *PALM3*, *AC022098.1*, *C19orf67*, *SAMD1*, *PRKACA*, *ASF1B*, *LPHN1*, *CD97*, *DDX39*, *PKN1*, *PTGER*, *GIPC1*, *DNAJB1*, *TECR*, *NDUFB7*, *CLECL17A*, *EMR3*) (Fig. 1). To test whether protein-disrupting mutations were present on the residual alleles, all coding exons and flanking intronic regions were sequenced by Sanger sequencing. Briefly, PCR primers were designed for each coding exon including at least 50 bp of flanking intronic sequences and are available upon request. Sequencing was performed on PCR products amplified from patient genomic DNA by SeqWright (Houston, TX). Variant Reporter software (Applied Biosystems, Foster City, CA) was used for variant calling and all intronic and coding variants identified were filtered on dbSNP build 131. A total of 44 novel variants were identified, mostly intronic and distant from the splice sites (35/44), but none of these variants was predicted to be pathogenic (Table I). Only one coding variant was not reported on dbSNP build 134 (http://www.ncbi.nlm.nih.gov/projects/SNP/): The p.S120L missense mutation within the *AC022098*.1 gene, encoding for a protein with unknown function (actually, on dbSNP “there is no contig mRNA transcript for this gene”).

**TABLE I:** List of Coding Variants Identified on the Residual Allele of Genes Mapping Within the 19p13.12–p13.13 Deleted Region

Gene	RefSeq	cDNA variant	Aminoacid substitution	dbSNP	PolyPhen-2	SIFT	Reported allele frequency
*RFX1*	NM_002918.4	c.1108 A > G	T370A	rs2305780	Benign (0.064)	Tolerated (0.15)	0.475^a^
*IL27RA*	NM_004843.2	c.21 C > T	A7A	rs113593439	n.a.	n.a.	0.958^a^
*AC022098.1*	(NR_024282.1)	c.359 C > G	S120W	no	Unknown	Damaging (0.04)	n.a.
*PKN1*	NM_002741.3	c.771 A > G	L257L	rs8107892	n.a.	n.a.	0.013^a^
		c.2772 C > T	L924L	rs8598	n.a.	n.a.	0.242^a^
*GIPC1*	NM_202468.1	c.852 G > A	A284A	rs1127307	n.a.	n.a.	0.200^a^
*EMR3*	NM_032571.3	c.379 C > G	E127Q	rs4606855	Benign (0.010)	Tolerated (0.2)	0.142^a^
		c.707 C > T	A236V	rs34226397	Benign (0.002)	Tolerated (1)	0.308^a^
		c.1154 G > A	R385Q	rs8102646	Benign (0.031)	Tolerated (1)	0.892^b^

n.a.: Not available.

a 1000 GENOMES:pilot 1 CEU low coverage panel.

b CORNELL:AGI_ASP_population.

By direct sequencing, we also screened both parents for the presence of this latter variant and we found that it was also carried by the propositus' mother, in heterozygous state, so we were able to conclude that the deletion originated on the paternal chromosome 19.

## DISCUSSION

Chromosome 19 is the most gene dense chromosome (about 24 protein coding genes per Mb, according to ENSEMBL database) of the human genome. Consequently, even small rearrangements could be lethal, explaining why very few of them are reported in the literature. Recently, with the development of molecular cytogenetic techniques, and particularly of genome-wide array analyses, there has been a modest increase in the number of described microdeletions and microduplications of small regions of chromosome 19.

Here, we report on a boy with a 1.2 Mb deletion on 19p13.12–p13.13 encompassing several genes (Fig. 1), including the first exon of the gene *CACNA1A*, whose mutations and large-scale gene rearrangements are responsible for episodic ataxia or familial hemiplegic migraine [Labrum et al., 2009]. Worthy of note are *CC2D1A* and *TECR*, whose mutations were recently discovered to be involved in non-syndromal autosomal recessive intellectual disability [Basel-Vanagaite et al., 2006; Çalışkan et al., 2011]. However, no mutations were found on the residual alleles, ruling out, in all probability that the phenotype we observed could belong to this category.

We found other five patients with partially overlapping 19p13.12–p13.13 microdeletions reported to date in the scientific literature. By comparing four of these cases (two new ones and those described by Engels et al., 2007 and by Jensen et al., 2009), Bonaglia et al. [2010] delineated a consistent phenotype and identified the corresponding smallest region of overlap (SRO), extending for about 360 kb and encompassing six annotated genes: *LPHN1*, *CD97*, *DDX39*, *PKN1*, *PTGER*, and *GIPC1*.

Phenotypic traits shared among these four patients were: Moderate to severe psychomotor delay, language delay, hyperactivity, hearing loss, congenital heart defects, and distinctive craniofacial features including brachycephaly, anteverted nares, and ear malformations. Low birthweight, short stature, microcephaly, brachydactyly, scoliosis were also common findings (Table II).

**TABLE II:** Clinical Features of Literature Patients With 19p13.12–p13.13 Deletions Overlapping With the Present Patient

Patient	Present patient	Bonaglia et al., 2010	Engels et al., 2007; Bonaglia et al., 2010	Bonaglia et al., 2010	Jensen et al., 2009	Van der Aa et al., 2010
		Patient 1	Patient 2	Patient 3		
Sex	m	m	f	m	f	m
At birth						
Weight	3rd–10th	−1.5 SD	3rd–10th	<3rd	<1st	<3rd
Length	25th	−1.5 SD	3rd	<3rd		<3rd
OFC	50th		−2.4 SD	10th–15th		3rd
At last evaluation						
Age (years)	7^3/12^	31	8^4/12^	7	10	15
Weight (kg) (%)	50th	+3 SD	75th	50th		50th
Length (cm) (%)	25th	−3 SD	3rd–10th	75th		3rd
OFC (cm) (%)	10th–25th	−1.3 SD	−3.2 SD	50th		
Hypotonia		−	−	+	−	
Intellectual disability	Mild	Moderate–severe	Moderate	Moderate–severe	Moderate	Moderate
Behavior	Hyperactivity, attention deficit	Hyperactivity, psychosis, auto- and hetero-aggressiveness	Hyperactivity	Hyperactivity		Shyness, insecurity, anxiety
Seizures/EEG anomalies	+	+		+		
Heart anomalies	−	Mild aortic and mitral vale incompetence	PDB, ASD, Bradycardia		ASD, VSD, PDB	−
Hearing loss	−	Sensorineural	Sensorineural	Conductive	Sensorineural	Conductive
Ocular anomalies		Myopia/nystagmus	Hyperopia, astigmatism		Strabismus	
Facial features	Upsweep of the frontal hairline	Brachycephaly	Brachycephaly	Brachycephaly	Tall forehead	Trigonocephaly
	Small ears with prominent anti-helix	Synophrys	Synophrys	Small mouth with thin upper lip	Occipital flattening	Widow's peak
	Mild hypertelorism	nodules, plaques, and erythema on the forehead	Almond-shaped eyes	Long philtrum	Telecanthus, ptosis	Bilateral frontal
	Highly arched palate	Arched eyebrows	Bilateral epicanthic fold	Anteverted ears	Epicanthal folds	Upsweep
		Mild blepharophimosis	Long eyelashes mild ptosis	Irregular teeth	Downslanting palpebral fissures	Synophrys
		Small/thin nasal root	Flattened nasal bridge	Bilateral epicanthic folds	Glabellar hemangioma	Thick eyebrows,
		Anteverted nares	Anteverted nostrils		Malar hypoplasia	Epicanthal folds,
		Anteverted ears with thin helices	Long philtrum		Short nose with anteverted nares	Flat nasal bridge
		Short neck	Small mouth with thin upper lip		Long philtrum,	Prominent incisors
					Flattened vermillion border	High palate
					Mild micrognathia.	Large ears
					Small, rounded, mildly low set auricles with pits on the lobules	Short neck
					Mild stenosis of the external auditory canals	
Other anomalies	Mild generalized hypertrichosis	Hypospadia	Hypodontia	Cryptorchidism	Scoliosis	Obesity
		Scoliosis		Scoliosis	Brachydactyly	Hypertrichosis
		Syndactyly		Irregular dentition	Hypodontia	Narrow brainstem
		Brachydactyly		Brachydactyly	Bilateral cervical sinuses	Gracile corpus callosum
		Obesity, hyperlipidemia			Eustachian tube dysfunction	Precocious puberty
		Hypertrichosis			Supernumerary nipples	
		Precocious puberty			Mild corpus callosum hypoplasia	
		Hypothyroidism,			Cerebellar vermis hypoplasia	
		Mild hepatic steatosis			Large cisterna magna and fourth ventricle	
		Submucous cleft palate				

Van der Aa et al. [2010] described a 1.2 Mb deletion involving the SRO, with only the *LPHN1* gene excluded (Table II).

Three additional subjects, with overlapping deletions, are reported in the DECIPHER database (http://decipher.sanger.ac.uk/) and another one in the ECARUCA database (http://agserver01.azn.nl:8080/ecaruca/ecaruca.jsp). Although our patient's deletion encompasses the entire SRO of Bonaglia et al. [2010], he shares very few traits with the other patients, presenting with mild developmental delay and behavior anomalies, showing no malformations and not suffering from hearing loss, ocular defects, heart anomalies, or scoliosis. His growth parameters and head circumference had always been within the normal range. Shared findings were hypertrichosis (present in Patient 1 of Bonaglia et al. [2010] and in the patient of Van der Aa et al. [2010]), bilateral frontal upsweep (like in the patient of Van der Aa et al. [2010]) and small ears with prominent anti-helix that, in our opinion, can be seen in photographs provided for Patient 3 of Bonaglia et al. [2010] and for the patient of Van der Aa et al. [2010].

The milder phenotype could be explained by the effect of variable expressivity, but some remarks are in order.

First, the deletion effects could be modulated by the presence of coding variants on the residual allele of the genes included in the deleted interval, such as non-synonymous SNPs, per se not pathogenic even in homozygous state. For instance, in the 1000 Genomes project browser (http://browser.1000genomes.org/index.html), 10, 9, 3, 10, 4, and 7 non-synonymous variants can be found within the coding sequences of *LPHN1*, *CD97*, *DDX39*, *PKN1*, *PTGER*, and *GIPC1*, respectively. Furthermore, population data available for some of these SNPs show a non-marginal prevalence among Europeans. It cannot be excluded that some of these variants may be responsible for the more severe phenotype of previously reported subjects.

Second, the deletions of Patient 1 of Bonaglia et al. [2010], the patient of Engels et al. [2007] (who corresponds to Patient 2 of Bonaglia et al. [2010]) and the patient of Jensen et al. [2009], share a larger region of overlap ranging from about 14 Mb to 16 Mb, that completely include also the 1.2 Mb deletion of the patient of Van der Aa et al. [2010], while the rearrangement of Patient 3 of Bonaglia et al. [2010] is more telomeric. So, deleted genes outside the SRO could be responsible for, or substantially influence, such features as more severe ID, facial dysmorphisms, microcephaly, short stature, heart defects, and sensorineural hearing loss, that are not found in our patient. In this sense, it is worth noting that Kosaki et al. [2011] recently reported a telomeric deletion of 760 kb, that is non-overlapping with that of our patient, but was completely included within the deleted intervals of patients described by Jensen et al. [2009], Engels et al. [2007], and Patient 1 of Bonaglia et al. [2010] (Fig. 1). These latter patients and that of Kosaki et al. [2011] shared various anomalies (bilateral cervical sinuses, micrognathia, hypodontia, dysmorphic auricles, ear pits or tags, stenosis of the external auditory canals, and sensorineural and conductive deafness), related to a defect of branchial arch development, suggesting that the putative locus for this defect may be included in this region.

Finally, the most consistent characteristics among all patients, apart from developmental delay, which was much milder in our patient, seem to be behavior anomalies, namely hyperactivity, anxiety and aggressiveness, with our patient presenting hyperactivity and attention deficit. None of the above behavioral traits were present in the intellectually normal parents.

Several genes in the deleted intervals could be good candidates to explain both ID and the behavioral phenotype, since they are involved in different neurobiological pathways and processes: *CC2D1A* (OMIM ID 610055), *TECR* (OMIM ID 610057), *RFX1* (OMIM ID 600006), *RLN3* (OMIM ID 606855), *PRKACA* (OMIM ID 601639), *PKN1* (OMIM ID 601032), LPHN1,*PTGER1* (OMIM ID 176802), and *GIPC1* (OMIM ID 605072).

Besides, episodes of febrile seizures and the EEG focal anomalies could be explained by the involvement of *CACNA1A* (OMIM ID 601011), deleted in patients reported by Auvin et al. [2009] (suffering from epilepsy with infantile spasms) and Lysy et al. [2009], (showing electroencephalographic anomalies). Actually, it should be considered that Bonaglia et al.'s [2010]] Patient 3, although suffering from generalized clonic seizures, was not deleted for *CACNA1A*.

In conclusion, we report on a new patient carrying a microdeletion involving chromosome region 19p13.12. His mild clinical presentation expands our knowledge of the phenotypic spectrum associated with haploinsufficiency of genes mapping in that region. The SRO previously defined by Bonaglia et al. [2010], which essentially includes mild ID and ADHD, should be considered with caution, given that a more severe phenotype in individual patients could depend on the extent of the deletion outside the SRO. However, new studies about the involved genes and additional patients will certainly help to define more accurately the genotype–phenotype correlations of the syndromic condition linked to 19p13 deletions.

In the meanwhile, if the reported deletion were to be found accidentally at amniocentesis, it would pose a very serious counseling problem, calling again attention to the great caution that must be exercised in applying Array-CGH analysis to prenatal diagnosis.

## References

[b1] Auvin S, Holder-Espinasse M, Lamblin MD, Andrieux J (2009). Array-CGH detection of a de novo 0.7-Mb deletion in 19p13.13 including CACNA1A associated with mental retardation and epilepsy with infantile spasms. Epilepsia.

[b2] Basel-Vanagaite L, Attia R, Yahav M, Ferland RJ, Anteki L, Walsh CA, Olender T, Straussberg R, Magal N, Taub E, Drasinover V, Alkelai A, Bercovich D, Rechavi G, Simon AJ, Shohat M (2006). The CC2D1A, a member of a new gene family with C2 domains, is involved in autosomal recessive nonsyndromic mental retardation. J Med Genet.

[b3] Bonaglia MC, Marelli S, Novara F, Commodaro S, Borgatti R, Minardo G, Memo L, Mangold E, Beri S, Zucca C, Brambilla D, Molteni M, Giorda R, Weber RG, Zuffardi O (2010). Genotype-phenotype relationship in three cases with overlapping 19p13.12 microdeletions. Eur J Hum Genet.

[b4] Çalışkan M, Chong JX, Uricchio L, Anderson R, Chen P, Sougnez C, Garimella K, Gabriel SB, Depristo MA, Shakir K, Matern D, Das S, Waggoner D, Nicolae DL, Ober C (2011). Exome sequencing reveals a novel mutation for autosomal recessive non-syndromic mental retardation in the TECR gene on chromosome 19p13. Hum Mol Genet.

[b5] Engels H, Brockschmidt A, Hoischen A, Landwehr C, Bosse K, Walldorf C, Toedt G, Radlwimmer B, Propping P, Lichter P, Weber RG (2007). DNA microarray analysis identifies candidate regions and genes in unexplained mental retardation. Neurology.

[b6] Jensen DR, Martin DM, Gebarski S, Sahoo T, Brundage EK, Chinault AC, Otto EA, Chaki M, Hildebrandt F, Cheung SW, Lesperance MM (2009). A novel chromosome 19p13.12 deletion in a child with multiple congenital anomalies. Am J Med Genet Part A.

[b7] Kosaki K, Saito H, Kosaki R, Torii C, Kishi K, Takahashi T (2011). Branchial arch defects and 19p13.12 microdeletion: Defining the critical region into a 0.8M base interval. Am J Med Genet Part A.

[b8] Labrum RW, Rajakulendran S, Graves TD, Eunson LH, Bevan R, Sweeney MG, Hammans SR, Tubridy N, Britton T, Carr LJ, Ostergaard JR, Kennedy CR, Al-Memar A, Kullmann DM, Schorge S, Temple K, Davis MB, Hanna MG (2009). Large scale calcium channel gene rearrangements in episodic ataxia and hemiplegic migraine: Implications for diagnostic testing. J Med Genet.

[b9] Lysy PA, Ravoet M, Wustefeld S, Bernard P, Nassogne MC, Wyns E, Sibille C (2009). A new case of syndromic craniosynostosis with cryptic 19p13.2-p13.13 deletion. Am J Med Genet Part A.

[b10] Malan V, Raoul O, Firth HV, Royer G, Turleau C, Bernheim A, Willatt L, Munnich A, Vekemans M, Lyonnet S, Cormier-Daire V, Colleaux L (2009). 19q13.11 deletion syndrome: A novel clinically recognizable genetic condition identified by array-CGH. J Med Genet.

[b11] Van der Aa N, Vandeweyer G, Kooy RF (2010). A boy with mental retardation, obesity and hypertrichosis caused by a microdeletion of 19p13.12. Eur J Med Genet.

